# Influence of an enhanced recovery programme on clinical outcomes and health-related quality of life after pancreaticoduodenectomy ad modum Whipple – an explorative and comparative single-centre study

**DOI:** 10.1186/s12893-024-02667-x

**Published:** 2024-12-21

**Authors:** Thomas Andersson, My Engström, Johanna Wennerblom, Hanna Gyllensten, Kristofer Bjerså

**Affiliations:** 1 Department of Surgery, Institute of Clinical Sciences, Sahlgrenska Academy, University of Gothenburg, Gothenburg, Sweden; 2 Region Västra Göteland, Department of Surgery, Sahlgrenska University Hospital, Gothenburg, Sweden; 3 Institute of Health and Care Sciences, Sahlgrenska Academy University of Gothenburg, Gothenburg, Sweden; 4 Family Medicine, School of Public Health and Community Medicine, Institute of Medicine, Sahlgrenska Academy, University of Gothenburg, Gothenburg, Sweden; 5 Region Västra Götaland, Primary Care, Närhälsan Majorna, Gothenburg, Sweden

**Keywords:** Enhanced recovery program, Pancreatic surgery, Health related quality of life

## Abstract

**Background:**

The introduction of enhanced recovery programmes (ERP) in pancreatic surgery has significantly improved clinical outcomes by decreasing the length of hospital stay, cost and complications without increasing readmissions and reoperations. To complement evidence on these outcomes, there is a need to explore patients’ perspectives of a structured ERP. Therefore, this study aimed to explore the health-related quality of life (HRQoL) of patients before and after implementing ERP in pancreaticoduodenectomy ad modum Whipple (PD) at a regional surgical centre.

**Method:**

This was an explorative and comparative single-centre study in Sweden. A prospective cohort receiving ERP was included between October 2019 and December 2022 (*n* = 73) and was compared with a retrospective pre-ERP cohort between October 2011 and December 2013 (*n* = 65). EQ-5D, the European Organization for Research and Treatment of Cancer (EORCT) Quality of Life Questionnaire Cancer 30 items (QOL-C30), and EORCT Quality of Life Questionnaire pancreatic cancer module (QOL-PAN26) were collected preoperatively and at three and six months postoperatively. Demographic and clinical variables were collected from patient charts. Complications were expressed using the Clavien-Dindo Classification and the Comprehensive Complications Index (CCI).

**Results:**

There were no significant differences in general health, cancer- or disease-specific HRQoL between the pre-ERP and ERP cohorts. Length of stay was significantly shorter in the ERP cohort (16 vs. 11 days; *p* < 0.001). There was no significant difference in CCI.

**Conclusion:**

No significant differences were found in the HRQoL of patients who participated in an ERP compared to those who did not. However, a significant decrease in LoS was found when ERP was applied.

**Trial registration:**

Not applicable.

**Supplementary Information:**

The online version contains supplementary material available at 10.1186/s12893-024-02667-x.

## Background

Enhanced recovery programmes (ERP) were introduced in the mid-nineties to improve recovery for surgical patients [1]. These programmes involve a multidisciplinary and multimodal approach to surgical care by structured use of evidence-based clinical interventions geared towards optimal and swift recovery during the pre-, peri-, and postoperative phases. Such interventions may include counselling and optimisation of present medical conditions, normovolemia, opioid-sparing analgesia, early return to per oral nutrition and early postoperative mobilisation. ERP have positive effects on clinical variables, such as decreasing length of stay (LoS), complications, and costs without increasing reoperations or readmissions [2].

While previous studies have provided evidence that clinical outcomes have improved after implementing ERP in pancreatic surgery, there are, to our knowledge, no studies examining patient-reported outcomes measures (PROM), such as health-related quality of life (HRQoL), in the evaluation of ERP within this type of surgery. Health has been defined by the World Health Organization (WHO) as *“a state of complete physical, mental and social well-being and not merely the absence of disease or infirmity”* [3]. The concept of health is interconnected with the concept of quality of life (QoL), which encompasses all aspects of life. HRQoL, on the other hand, refers specifically to the effects of illness and treatment on QoL [4]. According to Wilson and Cleary’s concept model [5], the HRQoL conceptual model can be divided into five levels, in which biological and physiological variables affect higher levels of outcome such as symptoms and functioning, and as an extension, overall health. Hence, as a multi-domain outcome, HRQoL is a relevant concept in evaluating advanced interventions such as ERP and can provide insights that can improve patient-centred care [6]. A previous review study on colorectal surgery patients showed no difference in HRQoL between groups that received standard care compared to ERP. Other studies have reported a faster return to daily activities and reduction of fatigue, but also higher levels of pain and lower emotional and mental health scores [7]. Two randomised controlled trials comparing ERP with standard care in gastric cancer surgery demonstrated shorter LoS but also improved HRQoL in the ERP cohorts [8, 9]. As ERP are consistently being implemented in pancreatic surgical care, there is a need to close the knowledge gap on how ERP impact the HRQoL of patients [10].

## Method

### Aim

The aim of this study was to explore surgical care outcomes including HRQoL of patients before and after implementing ERP in pancreaticoduodenectomy ad modum Whipple (hereafter PD) at a high- volume pancreatic unit. This study was performed as an explorative and comparative single-centre study at a university hospital and reported according to The Transparent Reporting of Evaluations with Nonrandomised Designs (TREND) [11].

### Samples and data collection

**Fig. 1:**
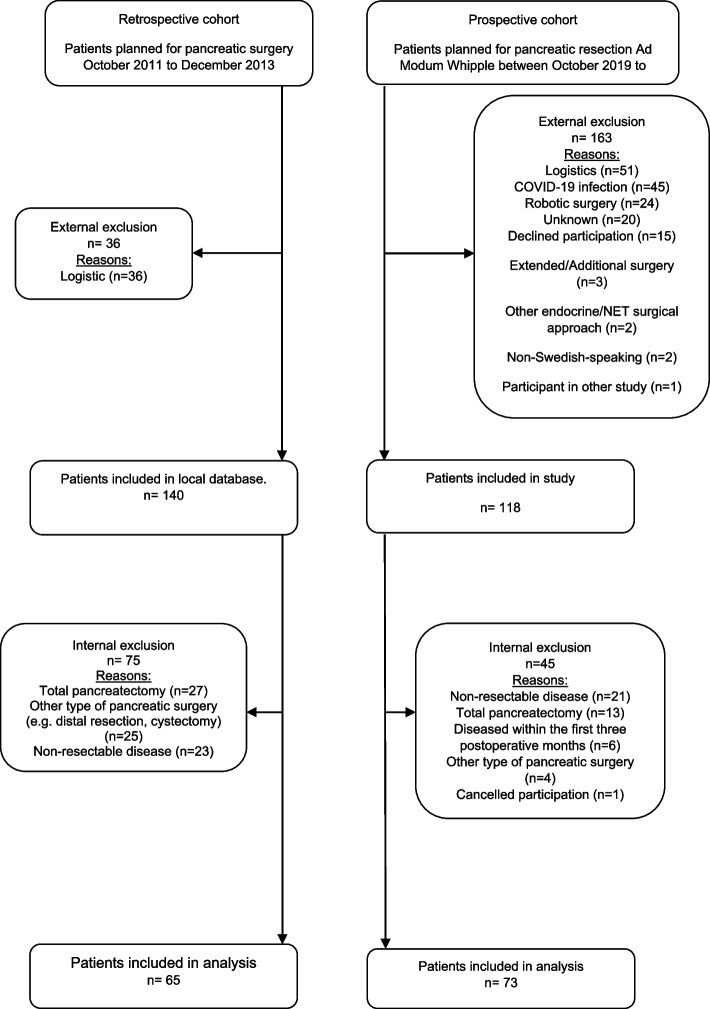
CONSORT Flow chart

Two cohorts of patients scheduled for pancreaticoduodenectomy (PD) at a university hospital in the west of Sweden were included in this study (Fig. 1). A retrospective pre-ERP cohort of patients from a clinical improvement project was included between October 2011 and December 2013, and a prospective cohort was included between October 2019 and December 2022. Patients were approached at the preoperative visit to request their participation, and upon enrolment, received questionnaires for baseline registration, postoperative follow up at was sent out and returned by mail. Inclusion criterion for the pre-ERP cohort was undergoing PD. Exclusion criteria were palliative resection due to metastasis or locally advanced disease, as well as additional or other types of pancreatic surgery. In the pre-ERP cohort, PROM together with additional clinical data were extracted from the medical records of all patients who underwent PD between October 2011 and December 2013. Minimal invasive procedures were excluded as these where not included in the ERP for pancreatic surgery at the time of the study.

In the ERP cohort, all patients scheduled for PD were approached at the preoperative visit to request their participation, and upon enrolment, received questionnaires for baseline registration. Postoperatively, the questionnaires together with return envelopes were sent out by post three and six months after surgery. Clinical data were extracted from medical records.

All data collection, including enrolment and logistics was conducted within our research group.

#### Measures

Disease-specific HRQoL: The European Organization for Research and Treatment of Cancer (EORCT) Quality of Life Questionnaire Cancer 30 items (QOL-C30), containing five functional scales, three symptom scales, a global health status scale, and six single-item scales [12] and EORCT Quality of Life Questionnaire pancreatic cancer module (QOL-PAN26), containing eight multi-item scales and 10 single items scales [13].

General HRQoL: The EQ-5D, consists of two components: a zero to 100 visual analogue scale (EQ VAS) to estimate general HRQoL at the time of response, in which a high score indicates better self-rated health; a descriptive scale measuring five dimensions of mobility, self-care, usual activities, pain/discomfort, and anxiety/depression. The responses to the dimensions are scored on three levels: 1 = no problems, 2 = some problems, 3 = extreme problems. A combination of these levels can be coded into 243 different states of health. Full health is indicated by 11,111 and the worst possible health by 33,333 [14]. The combination of responses to the five questions is further translated to utilities using both a society-based value set from the United Kingdom (UK) [15] and a Swedish experience-based value set [16] to enable analysis of general HRQoL.

#### Demographic and clinical variables

Preoperative variables: Age at inclusion, sex, comorbidities, American Society of Anaesthesiologists (ASA) classification [17], smoking, WHO performance status [18], involuntary weight loss, neoadjuvant chemotherapy. Perioperative variables: duration of surgical procedure (in minutes), duration of anaesthesia (in minutes), perioperative bleeding (in millilitres). Postoperative variables: diagnosis based on pathology (TNM), adjuvant chemotherapy, length of stay (in days), reoperations within primary stay, readmissions (30 and 60 days), complications (highest Clavien-Dindo classification) [19], comprehensive complication index (CCI) [20].

### Data analysis

Data were recorded using Microsoft Excel and analysed with IBM Statistical Package Social Science (SPSS) version 27, although for EQ-5D, utility translations were conducted using Stata Statistical Software: Release 17.0. College Station, TX: StataCorp LLC. Normal distribution was evaluated using histograms, and descriptive statistics were reported, including the mean, median, range, standard deviation (SD), and frequencies as percentages. Distribution differences, such as sex and smoking, were calculated using the Pearson Chi^2^ or Fischer’s exact test. Interval and frequency data were compared using the student’s t-test, and ordinal data by the Mann–Whitney U or the Friedman test. Confidence intervals were calculated for continuous clinical variables. After post hoc Bonferroni correction calculation, the level of statistical significance was set to p (α) < 0.001. A mixed between-within subject ANOVA was carried out on HRQoL measure occasions and group, Wilks’ Lambda was used on interaction effect (group differences together with measure occasions) as well as for measure occasion. Values for partial ETA squared according to Cohen [21] were used as the effect size variable: 0.01 = small effect, 0.06 = moderate effect, 0.14 = large effect size.

For the EORTC instruments and EQ-5D measures, individual missing items/rounds of responses were managed by excluding the calculated value in the hypothesised scales for each participant at a specific occasion. Only participants with both pre- and postoperative measures were included for analysis of change between measures. Analyses were conducted both for scales as continuous variables and results categorised as improved/unchanged/deteriorated. No imputation was conducted.

## Results

### Health-related quality of life

A total of 140 patients from the retrospective database and 118 from the prospective group were initially included, from these cohorts 73 prospective patients (ERP) were compared with 65 retrospective patients (Pre-ERP), see Table 1. Patient-rated general HRQoL, based on the EQ VAS and EQ-5D index scores, and cancer-specific HRQoL, based on the QOL-C30, were very similar between the pre-ERP and the ERP group at baseline (Tables 2 & 3). During the first three months, there was a trend of more patients improving in the pre-ERP cohort compared with the ERP. At six months, patients in the ERP cohort generally reported higher scores in both EQ VAS and EQ index scores. In terms of cancer-related HRQoL, mean QOL-C30 values were higher in the pre-ERP cohort compared to the ERP cohort at three months; also, more patients worsened in the ERP cohort compared with the pre-ERP cohort between baseline and three months. At six months the ERP cohort scored higher in global health status compared to the pre-ERP cohort; also, more patients improved in the ERP cohort between three and six months. However, the differences between the pre-ERP and ERP cohorts were not statistically significant (Table 2).

**Table 1:** Demographics and clinical variables for pre-ERP and ERP cohort

						**Standard surgical care (Pre-ERP)**	**ERP structured care (ERP)**	**P (95%CI.)**
Years of data collection						2011–2013	2019–2022	
Number of included patients form each cohort						*n* = 65	*n* = 73	
Demography	Sex (W/M)					49.2% / 50.7%	46.6% / 53.4%	0.755
	Age (mean (SD; min–max))					66.5 (9.0;44–80)	68.7 (11.4; 19–82)	0.211 (65.8–69.3)
	PreOp	Co-morbidity	Hypertension			24.6%	45.2%	0.012
			Diabetes			13.9%	23.3%	0.157
			Lung disease			6.2%	17.8%	0.045
			Kidney disease			0.0%	9.6%	0.010
			Cardiovascular			6.2%	27.4%	0.001
		Smokers				18.5%	9.6%	0.131
		Involuntary weight loss				43.1%	56.2%	0.197
		WHO performance status	Mean (SD)			0.7 (0.7)	0.5 (0.5)	
			Status 0			47.7%	48.6%	0.028
			Status 1			40.0%	50.0%	
			Status 2			12.3%	1.4%	
			Status 3			0%	0%	
			Status 4			0%	0%	
		ASA Class	Mean (SD)			1.7 (0.57)	2.2 (0.60)	
			ASA I			32.3%	9.7%	< 0.001
			ASA II			61.5%	59.7%	
			ASA III			6.2%	30.6%	
			ASA IV			0%	0%	
		Neoadjuvant chemotherapy				0%	5.6%	0.055
	IntraOp	Duration of anaesthesia in minutes				506(81;352–701)	551(113;358–850)	0.009 (512–546)
		Duration of surgical procedure in minutes				388(78;248–604)	437(110;278–754)	0.003 (397–430)
		Perioperative blood loss in millilitres				1084(730;200–4300)	484(408;50–2000)	< 0.001 (656–876)
		Vascular resections				7.6%	16.4%	
	PostOp	Diagnosis from PAD	Adenocarcinoma			89.2%	71.2%	
			IPNM			7.7%	9.6%	
			NET			1.5%	4.1%	
			Benign (tubulovillous adenoma, chronic inflammation, schwannoma)			0%	12.3%	
			Other			1.5%	2.7%	
		TNM classification when adenocarcinoma confirmed by pathology (Retrospective *n* = 58, Prospective *n* = 52)			T1	3.4%	13.5%	
					T2	19.0%	48.1%	
					T3	72.4%	34.6%	
					T4	5.2%	3.8%	
					N0	20.7%	25.0%	
					N1	79.3%	46.2%	
					N2	0.0%	28.8%	
					M0	100%	100%	
					M1	0.0%	0.0%	
		Adjuvant postoperative chemotherapy during the first 6 months				61.5%	49.3%	0.204
		Length of stay	Initial postoperative care at university hospital centre			16(7;5–33)	11(6;5–39)	< 0.001 (12–14)
			Total hospital stay including down stage care at general hospital			21(9;10–58)	18(7;7–61)	0.041 (17–20)
		Reoperation within primary hospital stay				4.7%	13.7%	0.067
		Readmission 30 days				14.1%	17.8%	0.526
		Readmission 60 days				12.5%	11.0%	0.805
		Complications	Clavien-Dindo Comprehensive complication index (CCI) (0–100)			18.8(13.7;0–58.4)	19.3(15.6;0–63.8)	0.636 (16.5–21.5)
			Highest Clavien-Dindo score prevalence in percent		No complication	30.8%	30.1%	
					1	9.2%	5.5%	
					2	50.8%	49.3%	
					3A	1.5%	1.4%	0.868
					3B	6.2%	11.0%	
					4A	1.5%	1.4%	
					4B	0.0%	1.4%	
					5	0.0%	0.0%	
			Pancreatic specific complications	Pancreatic fistula	B	6.2%	2.7%	
					C	0.0%	0.0%	
				Postoperative haemorrhage	A	1.5%	0.0%	
					B	3.1%	0.0%	
					C	1.5%	4.1%	
				Delayed gastric emptying	A	0.0%	1.4%	
					B	7.7%	9.6%	
					C	1.5%	0.0%	
			Postoperative mortality		Within 60 days	0.0%	3.0%	
					Within 90 days	3.1%	3.0%	
					Within 1 year	16.9%	15.0%

**Table 2:** EORTC QLQ C30 measurements

Scales	Cohort	Preoperative		Change preoperative to 3 months				3 months		Change 3 months to 6 months			
		Mean (SD;Min–Max)	p^A^	N of patients:			p^B^	Mean (SD;Min–Max)	p^A^	N of patients			p^B^
				Improved	Unchanged	Worsening				Improved	Unchanged	Worsening	
Global health status^a^	Pre-ERP	64 (25;0–100)(*n* = 55)	0.753	16	12	19	0.363	64 (24;0–100)(*n* = 54)	0.185	20	11	15	0.180
	ERP	65 (25;0–100)(*n* = 68)		16	11	32		57 (25;0–100)(*n* = 64)		31	7	12	
Physical functioning^b^	Pre-ERP	18(22;0–93) (*n* = 55)	0.704	26	11	11	0.507	23 (18;0–60) (*n* = 55)	0.343	16	12	18	0.104
	ERP	15(17;0–80)(*n* = 68)		37	11	9		27 (21;0–93)(*n* = 62)		8	14	27	
Role functioning^b^	Pre-ERP	32 (35;0–100) (*n* = 56)	0.988	19	16	14	0.682	33 (32; 0–100) (*n* = 55)	0.350	13	19	14	0.059
	ERP	30(30;0–100)(*n* = 68)		27	19	13		38 (32;0–100) (*n* = 64)		5	24	22	
Emotional functioning^2^	Pre-ERP	27 (21;0–100) (*n* = 54)	0.588	10	11	25	0.274	21 (20;0–83) (*n* = 54)	0.309	18	15	13	0.071
	ERP	26(23;0–83) (*n* = 70)		10	17	24		22 (21;0–83)(*n* = 64)		9	22	19	
Cognitive functioning^b^	Pre-ERP	14 (22;0–100) (*n* = 55)	0.510	13	26	8	0.355	15 (19;0–83) (*n* = 54)	0.762	13	24	9	0.621
	ERP	15 (21;0–83)(*n* = 68)		20	24	14		16 (19;0–83) (*n* = 62)		11	31	8	
Social functioning^b^	Pre-ERP	20 (26;0–100)(*n* = 55)	0.450	16	19	12	0.924	24 (23;0–100) (*n* = 46)	0.015	17	15	14	0.123
	ERP	24 (27;0–100)(*n* = 70)		23	23	15		30 (28;0–100) (*n* = 63)		9	22	18	
Fatigue^c^	Pre-ERP	35 (27;0–100)(*n* = 55)	0.584	15	7	24	0.258	40 (25;0–100) (*n* = 53)	0.260	18	10	17	0.123
	ERP	30 (22;0–88)(*n* = 67)		11	11	37		46 (28;0–100) (*n* = 63)		29	11	10	
Nausea and vomiting^c^	Pre-ERP	8 (17;0–100)(*n* = 56	0.701	8	23	18	0.621	13 (20;0–100) (*n* = 55)	0.239	8	28	11	0.296
	ERP	10 (21;0–100)(*n* = 68)		6	31	22		20 (28;0–100) (*n* = 63)		14	30	7	
Pain^c^	Pre-ERP	19 (24;0–100)(*n* = 55)	0.323	12	15	20	0.266	24 (23;0–100) (*n* = 54)	0.016	14	24	8	0.721
	ERP	14 (21;0–66) (*n* = 69)		14	28	18		16 (24;0–100) (*n* = 63)		12	30	8	
Dyspnoea^c^	Pre-ERP	16 (20;0–66)(*n* = 55)	0.311	4	31	13	0.589	22 (21;0–67) (*n* = 55)	0.709	8	29	10	0.438
	ERP	20 (23;0–100)(*n* = 70)		9	37	15		24 (26;0–67) (*n* = 64)		9	36	6	
Insomnia^c^	Pre-ERP	33 (33;0–100)(*n* = 56)	0.938	17	20	12	0.340	32 (31;0–100) (*n* = 55)	0.034	13	23	11	0.377
	ERP	32 (31;0–100) (*n* = 70)		20	32	9		21 (29;0–100) (*n* = 64)		11	32	8	
Appetite loss^c^	Pre-ERP	16 (25;0–100)(*n* = 56)	0.022	11	23	15	0.347	24 (30;0–100) (*n* = 54)	0.056	11	28	8	0.508
	ERP	28 (31;0–100)(*n* = 70)		14	21	26		37 (37;0–100) (*n* = 64)		17	25	9	
Constipation^c^	Pre-ERP	10 (21;0–100)(*n* = 56)	0.045	7	34	8	0.394	9 (18;0–67) (*n* = 55)	0.102	3	38	6	0.235
	ERP	18 (27;0–100)(*n* = 70)		14	35	12		17 (27;0–100) (*n* = 64)		9	36	6	
Diarrhoea^c^	Pre-ERP	16 (27;0–100)(*n* = 55)	0.274	6	29	12	0.402	22 (28;0–100) (*n* = 54)	0.615	7	25	15	0.854
	ERP	10 (21;0–100)(*n* = 70)		6	32	23		27 (35;0–100) (*n* = 63)		8	29	14	
Finance difficulties^c^	Pre-ERP	6 (16;0–66)(*n* = 55)	0.797	4	38	5	0.218	7 (18;0–67) (*n* = 54)	0.201	4	37	5	0.187
	ERP	6 (16;0–66)(*n* = 69)		4	50	9		13 (24;0–100) (*n* = 64)		6	43	1	

Scales	6 months		Change preoperative to 6 months			
	Mean (SD;Min–Max)	p^A^	N of patients			p^B^
			Improved	Unchanged	Worsening	
Global health status^a^	62 (24;0–100) (*n* = 50)	0.124	17	5	20	0.877
	69 (22;8–100) (*n* = 57)		21	8	23	
Physical functioning^b^	24 (20;0–73) (*n* = 49)	0.193	25	6	10	0.210
	19 (18;0–80.0) (*n* = 57)		29	15	8	
Role functioning^b^	34 (27;0–83) (*n* = 49)	0.062	19	11	12	0.528
	26 (29;0–100) (*n* = 57)		18	16	19	
Emotional functioning^2^	24 (20;0–92) (*n* = 50)	0.003	12	8	22	0.642
	14 (20;0–75.0) (*n* = 50)		11	12	31	
Cognitive functioning^b^	18 (22;0–67) (*n* = 50)	0.869	17	15	10	0.529
	17 (22;0–100) (*n* = 58)		18	25	10	
Social functioning^b^	28 (23;0–83) (*n* = 50)	0.067	18	16	8	0.319
	22 (27;0–100) (*n* = 55)		17	19	17	
Fatigue^c^	43 (25;0–100) (*n* = 50)	0.050	12	9	21	0.994
	35 (26;0–100) (*n* = 58)		15	12	27	
Nausea and vomiting^c^	16(23;0–100) (*n* = 50)	0.184	5	21	17	0.220
	10 (14;0–50.0) (*n* = 58)		12	29	14	
Pain^c^	21 (25;0–100) (*n* = 50)	0.032	8	23	11	0.724
	13 (25;0–100) (*n* = 58)		13	31	11	
Dyspnoea^c^	25 (27;0–100) (*n* = 50)	0.284	2	27	13	0.144
	20 (23;0–67) (*n* = 58)		8	37	10	
Insomnia^c^	33 (30;0–100) (*n* = 50)	0.054	13	19	11	0.385
	23 (30;0–100) (*n* = 58)		20	27	8	
Appetite loss^c^	22 (32;0–100) (*n* = 50)	0.457	9	20	14	0.431
	24 (30;0–100) (*n* = 58)		18	22	15	
Constipation^c^	12 (20;0–67) (*n* = 50)	0.969	6	30	7	0.129
	14 (25;0–100) (*n* = 58)		16	28	11	
Diarrhoea^c^	29 (29;0–100) (*n* = 50)	0.639	6	14	22	0.232
	27 (29;0–100) (*n* = 58)		4	27	24	
Finance difficulties^c^	11 (24;0–100) (*n* = 50)	0.341	4	32	6	0.405
	6 (17;0–100) (*n* = 57)		3	46	4	

^A^Mann-Whitney U test

^B^Chi^2^-test

^a^High score represents high quality of life

^b^High score represents high level of functioning

^c^High score represents high level of symptomatology and problems

**Table 3:** EQ5D measured preoperatively, at 3 months and 6 months, and difference between each occasion

	Cohort	Preoperative		Change preoperative to 3 months				3 months		Change 3 months to 6 months			
		Mean(SD; Min–Max	p^A^	N of patients:			p^B^	Mean(SD; Min–Max	p^A^	N of patients:			p^B^
				Improved	Unchanged	Worsening				Improved	Unchanged	Worsening	
EQ VAS	Pre-ERP	58 (30;0–100)(*n* = 55)	0.211	29	0	19	0.08	65 (25;0–100)(*n* = 55)	0.825	24	5	20	0.171
	ERP	67 (23;5–100)(*n* = 59)		24	4	25		65 (23;18–100)(*n* = 63)		33	4	12	
EQ INDEX Swedish Experienced based	Pre-ERP	0.86 (0.13;0.34–0.97) (*n* = 56)	0.320	26	8	15	0.067	0.87 (0.11;0.55–0.97) (*n* = 55)	0.111	14	15	19	0.019
	ERP	0.85 (0.11;0.52–0.97) (*n* = 67)		19	14	28		0.85 (0.12;0.42–0.97) (*n* = 66)		25	19	8	
EQ INDEX UK Society based	Pre-ERP	0.73 (0.29;-0.48–1.00) (*n* = 56)	0.823	24	8	17	0.295	0.75 (0.24;0.09–1.0) (*n* = 55)	0.787	16	15	17	0.396
	ERP	0.73 (0.24;-0.01–1.0) (*n* = 67)		21	14	26		0.73 (0.23;-0.12–1.0) (*n* = 66)		21	19	12	

	6 months		Change preoperative to 6 months			
	Mean(SD;Min–Max	p^A^	N of patients:			p^B^
			Improved	Unchanged	Worsening	
EQ VAS	64 (27;0–100)(*n* = 52)	0.030	23	7	14	0.245
	75 (20;19–100)(*n* = 57)		31	3	12	
EQ INDEX Swedish Experienced based	0.86 (0.10;0.49–0.97) (*n* = 51)	0.052	18	8	18	0.619
	0.89 (0.09;0.61–0.97) (*n* = 57)		22	13	17	
EQ INDEX UK Society based	0.76 (0.22;-0.32–1.0) (*n* = 51)	0.171	16	8	20	0.422
	0.81 (0.19;0.09–1.0) (*n* = 57)		22	13	17	

^A^Mann-Whitney U test

^B^Chi^2^-test

Functioning scale scores of the QOL-C30 and QLQ-PAN26 were not significantly different between the ERP and pre-ERP cohorts (Tables 2 & 4). There was a trend of slightly higher or similar scores in the ERP cohort at baseline and at three months. However, at six months, the trend was reversed, with higher functional scale scores in the pre-ERP cohort and more patients had worsening or unchanged scores in functional scales over time in the ERP cohort. Satisfaction with health care scores was highest preoperatively and deteriorated over time in both cohorts, with more patients having worsening or unchanged scores. Overall, ERP care was not better than pre-ERP care in terms of functional scale scores.

**Table 4:** EORTC QLQ PAN26 measured preoperatively, at 3 months and 6 months, and difference between each occasion

Scales	Cohort	Preoperative		Change preoperative to 3 months				3 months		Change 3 months to 6 months			
		Mean (SD;Min–Max)	p^A^	N of patients:			p^B^	Mean (SD;Min–Max)	p^A^	N of patients			p^B^
				Improved	Unchanged	Worsening				Improved	Unchanged	Worsening	
Pancreatic pain^a^	Pre-ERP	18 (18;0–67)(*n* = 52)	0.601	20	13	13	0.992	22 (21;0–83)(*n* = 54)	0.771	16	13	18	0.013
	ERP	18 (21;0–100)(*n* = 67)		28	18	17		20(18;0–83)(*n* = 65)		18	25	7	
Bloating^a^	Pre-ERP	21 (26;0–100)(*n* = 52)	0.272	15	24	7	0.641	32 (30;0–100)(*n* = 54)	0.010	12	20	16	0.027
	ERP	17 (25;0–100)(*n* = 69)		16	32	13		20 (26;0–100)(*n* = 65)		10	24	7	
Digestive symptoms^a^	Pre-ERP	16 (24;0–100)(*n* = 52)	0.190	26	15	5	0.274	32 (27;0–100)(*n* = 53)	0.213	20	16	12	0.477
	ERP	22 (28;0–100)(*n* = 69)		36	13	12		39 (30;0–100)(*n* = 64)		22	21	8	
Taste^a^	Pre-ERP	18 (28;0–100)(*n* = 52)	0.512	29	19	7	0.494	33 (33;0–100)(*n* = 54)	0.057	13	29	6	0.744
	ERP	21 (28;0–100)(*n* = 69)		33	22	6		46 (38;0–100)(*n* = 65)		17	27	7	
Indigestion^a^	Pre-ERP	17 (28;0–100)(*n* = 52)	0.757	16	22	8	0.716	24 (26;0–100)(*n* = 54)	0.404	10	22	16	0.196
	ERP	17 (27;0–100)(*n* = 66)		23	28	7		30 (32;0–100)(*n* = 63)		14	28	9	
Flatulence^a^	Pre-ERP	29 (35;0–100)(*n* = 52)	0.872	20	22	4	0.931	45 (36;0–100)(*n* = 54)	0.526	11	21	16	0.978
	ERP	26 (27;0–100)(*n* = 69)		28	27	6		41 (33;0–100)(*n* = 65)		12	23	16	
Weight loss	Pre-ERP	24 (32;0–100)(*n* = 52)	0.467	17	22	6	0.504	39 (36;0–100)(*n* = 53)	0.778	14	28	6	0.981
	ERP	20 (29;0–100)(*n* = 69)		29	23	9		41 (37;0–100)(*n* = 65)		15	29	7	
Weakness in arms and legs^a^	Pre-ERP	19 (26;0–100)(*n* = 52)	0.745	19	22	5	0.951	33 (29;0–100)(*n* = 54)	0.596	12	22	14	0.463
	ERP	19 (22;0–67)(*n* = 69)		27	28	6		40 (33;0–100)(*n* = 65)		12	29	10	
Dry mouth^a^	Pre-ERP	26 (31;0–100)(*n* = 52)	0.648	11	26	9	0.538	24 (28;0–100)(*n* = 54)	0.951	9	28	11	0.808
	ERP	25 (34:0–100)(*n* = 69)		17	28	16		25 (31;0–100)(*n* = 65)		10	33	8	
Hepatic symptoms^a^	Pre-ERP	23 (34;0–100)(*n* = 51)	0.799	6	19	21	0.239	7 (16;0–83)(*n* = 54)	0.110	5	39	4	0.182
	ERP	20 (30;0–100)(*n* = 68)		3	32	25		4 (11;0–50)(*n* = 65)		4	36	11	
Altered bowel habits^a^	Pre-ERP	21 (26;0–100)(*n* = 52)	0.854	20	17	8	0.375	29 (28;0–100)(*n* = 53)	0.816	14	12	21	0.318
	ERP	19 (22;0–100)(*n* = 65)		29	14	13		31 (30;0–100)(*n* = 64)		12	20	18	
Body image^a^	Pre-ERP	16 (23;0–100)(*n* = 51)	0.647	18	19	9	0.684	24 (26;0–100)(*n* = 54)	0.381	12	22	14	0.069
	ERP	19 (24;0–100)(*n* = 65)		26	22	8		28 (28;0–100)(*n* = 64)		16	27	5	
Troubled with side-effects^a^	Pre-ERP	17 (27;0–67)(*n* = 52)	0.995	24	18	4	0.423	41 (32;0–100)(*n* = 54)	0.766	15	24	8	0.757
	ERP	15 (23;0–67)(*n* = 66)		38	18	3		40 (28;0–100)(*n* = 66)		17	28	6	
Future worries^a^	Pre-ERP	53 (27;0–100)(*n* = 52)	0.813	5	24	17	0.463	44 (30;0–100)(*n* = 54)	0.725	9	30	8	0.425
	ERP	54 (32;0–100)(*n* = 69)		12	28	21		47 (31;0–100)(*n* = 66)		15	29	6	
Planning of activities^a^	Pre-ERP	32 (29;0–100)(*n* = 51)	0.877	14	22	9	0.212	33 (31;0–100)(*n* = 54)	0.809	13	24	9	0.561
	ERP	34 (33;0–100)(*n* = 69)		22	29	19		33 (34;0–100)(*n* = 66)		15	30	6	
Satisfaction with health care^b^	Pre-ERP	78 (27;0–100)(*n* = 50)	0.817	18	10	17	0.123	76 (27;0–100)(*n* = 53)	0.006	14	11	18	0.828
	ERP	75 (30;0–100)(*n* = 64)		12	12	30		62 (28;0–100)(*n* = 64)		15	15	18	
Sexuality^b^	Pre-ERP	45 (42;0–100)(*n* = 48)	0.842	14	17	10	0.274	54 (37;0–100)(*n* = 49)	0.890	8	17	14	0.623
	ERP	43 (37;0–100)(*n* = 59)		25	15	9		56 (37;0–100)(*n* = 57)		9	15	20	

Scales	6 months		Change preoperative to 6 months			
	Mean (SD;Min–Max)	p^A^	N of patients			p^B^
			Improved	Unchanged	Worsening	
Pancreatic pain^a^	24 (20;0–75)(*n* = 51)	0.007	11	11	20	0.132
	15 (17;0–75)(*n* = 56)		18	19	14	
Bloating^a^	35 (31;0–100)(*n* = 52)	0.009	5	17	21	0.029
	16 (24;0–100)(*n* = 57)		14	26	13	
Digestive symptoms^a^	31 (27;0–100)(*n* = 52)	0.292	17	15	21	0.606
	26 (26;0–100)(*n* = 57)		13	16	24	
Taste^a^	24 (30;0–100)(*n* = 52)	0.113	10	18	15	0.478
	35 (35;0–100)(*n* = 57)		10	18	25	
Indigestion^a^	31 (33;0–100)(*n* = 52)	0.333	7	20	16	0.734
	26 (31;0–100)(*n* = 57)		8	27	15	
Flatulence^a^	49 (34;0–100)(*n* = 52)	0.221	6	14	23	0.656
	41 (32;0–100)(*n* = 57)		7	22	24	
Weight loss	31 (37;0–100)(*n* = 52)	0.768	10	22	11	0.673
	28 (34;0–100)(*n* = 57)		11	24	18	
Weakness in arms and legs^a^	39 (38;0–100)(*n* = 52)	0.228	4	22	17	0.667
	29 (33;0–100)(*n* = 57)		8	24	21	
Dry mouth^a^	24 (28;0–100)(*n* = 52)	0.860	11	21	11	0.991
	23 (29;0–100)(*n* = 57)		14	26	13	
Hepatic symptoms^a^	5 (12;0–50)(*n* = 52)	0.756	20	17	5	0.692
	5 (10;0–50)(*n* = 57)		21	26	6	
Altered bowel habits^a^	32 (29;0–100)(*n* = 52)	0.790	10	8	25	0.716
	31 (28;0–100)(*n* = 56)		13	12	25	
Body image^a^	28 (30;0–100)(*n* = 52)	0.084	7	14	21	0.095
	19 (27;0–100)(*n* = 55)		15	19	14	
Troubled with side-effects^a^	33 (29;0–100)(*n* = 52)	0.526	7	14	22	0.467
	29 (25;0–100)(*n* = 56)		4	19	26	
Future worries^a^	43 (31;0–100)(*n* = 52)	0.256	15	21	7	0.126
	36 (29;0–100)(*n* = 56)		29	17	6	
Planning of activities^a^	33 (29;0–100)(*n* = 51)	0.157	10	22	9	0.353
	24 (27;0–100)(*n* = 57)		20	23	9	
Satisfaction with health care^b^	66 (29;0–100)(*n* = 49)	0.156	12	6	22	0.201
	56 (36;0–100)(*n* = 55)		7	11	29	
Sexuality^b^	48 (36;0–100)(*n* = 47)	0.001	13	12	13	0.779
	42 (34;0–100)(*n* = 50)		12	16	13	

^A^Mann-Whitney U test

^B^Chi^2^-test

^a^High score represents high level of symptomatology and problems

^b^High score represents high level of functioning

Symptom scale scores of the QOL-C30 and QOL-PAN26 were not significantly different between the ERP and pre-ERP cohorts (Tables 2 & 4). At three months the ERP cohort scored higher in more symptom scales compared with the pre-ERP cohort. The overall symptom burden remained high at six months compared with preoperative measurements in both cohorts. Also, at six months there was a trend of less symptom burden in the ERP cohort compared with the pre-ERP cohort.

The mixed between-within subject ANOVA did not show any interaction effect between intervention and time of measurement (Table 5). There was no significant interaction effect between ERP and measure occasion. There was a measure occasion effect for diarrhoea in QOL C30 and for digestive symptoms, taste, flatulence, weight loss, weakness in the arms and legs, hepatic symptoms, and trouble with side-effects in QOL PAN26 over the three measurement times in symptom scales. There was no significant group effect, indicating no effect from ERP in these results.

**Table 5:** Mixed between-within subject ANOVA for HRQoL outcomes measure

		**Interaction effect** (group x measure occasion)		**Group effect** (Pre-ERP and ERP)		**Measure occasion effect** (Preoperative, 3 months, 6 months)	
		Effect size^1^	P^2^	Effect size^1^	P	Effect size^1^	P^2^
EQ5D	EQ VAS	0.136	0.003	0.013	0.307	0.062	0.074
	EQ INDEX- Swedish Experienced based	0.050	0.103	0.001	0.813	0.028	0.290
	EQ INDEX—UK Society based	0.009	0.675	0.003	0.584	0.048	0.115
EORTC C30	Global health status	0.081	0.031	0.006	0.492	0.033	0.256
	Physical functioning	0.068	0.057	0.018	0.225	0.135	0.003
	Role functioning	0.057	0.082	0.021	0.173	0.032	0.252
	Emotional functioning	0.036	0.213	0.042	0.055	0.116	0.006
	Cognitive functioning	0.004	0.864	0.001	0.835	0.045	0.152
	Social functioning	0.050	0.114	0.001	0.967	0.028	0.298
	Fatigue	0.075	0.041	0.013	0.291	0.083	0.029
	Nausea and vomiting	0.091	0.016	0.008	0.404	0.067	0.049
	Pain	0.001	0.993	0.082	0.007	0.035	0.228
	Dyspnoea	0.048	0.122	0.015	0.255	0.066	0.052
	Insomnia	0.012	0.591	0.040	0.057	0.063	0.060
	Appetite loss	0.044	0.142	0.007	0.423	0.025	0.338
	Constipation	0.030	0.262	0.006	0.458	0.005	0.794
	Diarrhoea	0.001	0.996	0.005	0.506	0.189	** < 0.001**
	Finance difficulties	0.031	0.268	0.001	0.910	0.062	0.072
EORTC PAN26	Pancreatic pain	0.036	0.218	0.037	0.078	0.024	0.370
	Bloating	0.058	0.077	0.079	0.008	0.067	0.052
	Digestive symptoms	0.014	0.548	0.001	0.793	0.207	** < 0.001**
	Taste	0.032	0.255	0.015	0.260	0.231	** < 0.001**
	Indigestion	0.040	0.189	0.001	0.800	0.116	0.006
	Flatulence	0.004	0.855	0.016	0.238	0.271	** < 0.001**
	Weight loss	0.030	0.276	0.001	0.809	0.156	** < 0.001**
	Weakness in arms and legs	0.015	0.527	0.008	0.417	0.169	** < 0.001**
	Dry mouth	0.001	0.999	0.003	0.626	0.005	0.825
	Hepatic symptoms	0.036	0.210	0.010	0.357	0.265	** < 0.001**
	Altered bowel habits	0.002	0.929	0.001	0.916	0.148	0.002
	Body image	0.081	0.036	0.001	0.850	0.085	0.029
	Troubled with side-effects	0.002	0.903	0.031	0.104	0.386	** < 0.001**
	Future worries	0.026	0.316	0.008	0.396	0.128	0.003
	Planning of activities	0.035	0.224	0.002	0.655	0.030	0.271
	Satisfaction with health care	0.038	0.220	0.049	0.047	0.132	0.004
	Sexuality	0.014	0.620	0.001	0.967	0.071	0.075

^1^Effect size calculated by Partial Eta Squared

^2^*P*-value calculated for Wilks’ Lambda

### Clinical variables

There were no significant differences in patient distribution between the pre-ERP and ERP cohorts in terms of age and sex (Table 1). However, there was a higher proportion of adenocarcinoma patients in the pre-ERP cohort, while a higher proportion of patients in the ERP cohort had a benign diagnosis. Simultaneously, the proportion of intraductal papillary mucinous neoplasms (IPMN) was comparable between the cohorts. All compared comorbidities, except for diabetes, were significantly more common in the ERP cohort. Furthermore, the ERP cohort had an overall lower physical status as measured by ASA scoring. The pre-ERP cohort experienced more perioperative bleeding, while the duration of anaesthesia and surgery was significantly longer in the ERP cohort. The ERP cohort had a significantly shorter LoS at the surgical centre and total LoS. There were no significant differences between the two cohorts in CCI, reoperations, or readmissions at 30 or 60 days.

## Discussion

There is a lack of data on HRQoL in studies evaluating the effect on clinical outcomes of ERP in patients who have undergone PD. This is the first study to address the long-term effects (beyond 30 days) of ERP on general and disease-specific HRQoL after pancreaticoduodenectomy and the results shows a significanty shorter LoS in the ERP cohort without compromising HRQoL.

There were no significant differences in general and disease-specific HRQoL between pre-ERP and ERP cohorts. Patients’ HRQoL deteriorated at the three-month measurements in both cohorts but improved at the six-month measurements, returning to baseline measurements or even surpassing them slightly. Functioning scales measured at baseline were similar in both cohorts and there was an overall improvement at three months compared to baseline. However, at six months, functioning scales had deteriorated or remained unchanged compared to baseline with a trend of improvement in the pre-ERP cohort. This raises the question on how neoadjuvant chemotherapy, preoperative ASA score, comorbidities and vascular resections impact functional scores. This needs to be addressed in future multicentre studies.

Concerning disease-specific symptom burden, increased levels were observed at three and six months in both cohorts compared to baseline. However, here there was a positive trend in the ERP cohort, scoring generally lower in symptom-specific scales at six months compared to the pre-ERP cohort. The reason for this is unknown. ERPs typically focus on care at pre-admission, as well as during early and intermediate postoperative phase and not the late postoperative phase. According to Wilson and Cleary’s conceptual model on HRQoL [5], individual and environmental factors influence symptoms, functional status, and general health perception, which ERPs aim to address. Still, the effect of ERP on long term postoperative HRQoL needs to be further explored.

As for the decline in satisfaction with health care (Table 4), observed in both cohorts but to a greater extent in the ERP cohort, this might be related to that patients may struggle with their recovery on their own after discharge. Especially in the ERP cohort where LoS was shorter. Hence, patients may be prepared within the ERP for a declining function as well as increased symptom burden, but are still in need of support from formal and informal caregivers to mitigate effect on recovery which has described in qualitative studies [22, 23].

The pattern of patients regaining HRQoL after pancreatic surgery has been described in previous research. In a systematic review, physical, social, and global health status scales deteriorated during the first three months. However, after six months, the scales showed a return to baseline scores. Symptoms such as fatigue returned to baseline, diarrhoea worsened and pain was undetermined [24]. The present study describes a similar pattern within the global health status as well as functional and symptom scales. However, except from the trends discussed above, there were no significant differences between the pre-ERP and ERP cohorts. This lack of association with the implementation of ERP was also confirmed by the mixed between-within subject ANOVA, suggesting that ERP do not affect patient-reported HRQoL to any significant extent. However, there was a trend of better general health and HRQoL in the ERP cohort, which was confirmed in a recent systematic review [10] stating that ERP may have a positive impact in hepato-pancreatico-biliary surgery seven days postoperatively. However, in that study there were no measuring points beyond 30 days postoperatively.

The ERP cohort had a significantly longer operation time, which could be explained by the surgery being more advanced, patients being more physically impaired and higher proportion of vascular resections compared with the pre-ERP cohort (Table 1). This was confirmed in previous studies, stating that ASA classification > 3, preoperative chemotherapy, pancreatic duct < 3 mm in diameter, T-stage > 3 and vascular resection are risk factors for prolonged operating time and length of stay [25]. Length of hospital stay (LoS) has often been the primary variable for the evaluation of ERP in previous research, demonstrating a general decrease in LoS when ERP is implemented in pancreatic surgery [2].This is also confirmed in the present study, as the ERP cohort had a significantly shorter LoS, both at the primary surgical centre and in total, including hospital stay at a regional hospital before discharge. Additionally, current research indicate a strong correlation between LoS and complication rates measured by CCI in patients undergoing PD [26]. In our study, we found no significant difference in either CCI or readmission between the pre-ERP and the ERP cohort even though the ERP cohort had a significantly shorter LoS. This may indicate that other factors then postoperative complication burden alone is more related to LoS when applying ERP.

Patients in the ERP group were significantly more affected by comorbidities and had a significantly higher ASA score, which might generate a higher risk of complications [27–29]. However, there was no significant difference in CCI or the highest Clavien-Dindo Classification between the pre-ERP and the ERP cohort. According to Swedish national statistics, patients offered pancreatic surgery tend to be more physically impaired and with more comorbidities over the years [30]. There were more patients with benign disease in the ERP cohort. Other international studies also describe that about 10% of patients undergoing surgery for malignant or IPMN turns out to be benign [31].

This study has several limitations. Over time care changes and evolves such as surgical approach and staff turnover as well as the introduction of ERP (Supplement 1). During the data collection of both cohorts in this study the surgical team, as well as the facilities and logistics remained constant. Less visible is the change in care culture that the introduction of ERP brings. This culture change includes not only accepting new evidence but also an improved collaboration between disciplines and departments involved in the patients surgical journey. One confounding factor in the present study is to what extent patients and staff were compliant to the ERP. Unfortunately, there was no data available on this. Another confounding factor is that most patients finalize their hospital stay at other hospitals with different routines. This might have an impact on total LoS, or patient follow up after discharge. Within this study all data was collected from one surgical centre and the sample size must be assessed as small. The retrospective data collection in the pre-ERP cohort was subject to selection bias as lesser benign lesions and proportion of vascular resections, as well as more extensive growth according to TNM classification. One inherent problem with HRQoL data is the risk of response shift; some patients might subjectively adapt to a new level of functioning even though their objective, actual state remained unchanged. This might have influenced the result in the present study since as time passes, patients adapt and score higher in functional measurements or HRQoL than what is objectively true [32].

## Conclusion

No significant differences were found in the HRQoL of patients who participated in an ERP compared to those who did not. However, a significant decrease in LoS was found when ERP was applied.

## Supplementary Information

Supplementary Material 1.

## Data Availability

Datasets used during the current study are available from the corresponding author upon reasonable request.

## Abbreviations

ERP: Enhanced recovery programs
HRQoL: Health related quality of life
PD: Pancreaticoduodenectomy ad modum
EORTC: European Organization for Research and Treatment of Cancer
QOL-C30: Quality of Life Questionnaire Cancer 30 items
QOL-PAN26: Quality of Life Questionnaire pancreatic cancer module
CCI: Comprehensive Complications Index
PROM: Patient-reported outcomes measures
WHO: World Health Organization
QoL: Quality of life
TREND: The Transparent Reporting of Evaluations with Nonrandomised Designs
U.K: United Kingdom
SPSS: IBM Statistical Package Social Science
ASA: American Society of Anaesthesiologists
IPMN: Intraductal papillary mucinous neoplasms

## References

[1] Kehlet H. Multimodal approach to control postoperative pathophysiology and rehabilitation. Br J Anaesth. 1997;78(5):606–17. 9175983 10.1093/bja/78.5.606

[2] Kuemmerli C, Tschuor C, Kasai M, Alseidi AA, Balzano G, Bouwense S, et al. Impact of enhanced recovery protocols after pancreatoduodenectomy: meta-analysis. Br J Surg. 2022;109(3):256–66. 35037019 10.1093/bjs/znab436

[3] Organization WH. Constitution of the world health organization; 1995.

[4] Guyatt GH, Ferrans CE, Halyard MY, Revicki DA, Symonds TL, Varricchio CG, Kotzava A, Valderas JM, Alonso JL (2007 Exploration of the value of health-related quality-of-life information from clinical research and into clinical practice. Mayo Clinic Proceedings; 82:1229-39 10.4065/82.10.1229 17908529

[5] Wilson IB, Cleary PD. Linking clinical variables with health-related quality of life: a conceptual model of patient outcomes. JAMA. 1995;273(1):59–65. 7996652

[6] Kingsley C, Patel S. Patient-reported outcome measures and patient-reported experience measures. Bja Education. 2017;17(4):137–44.

[7] Li D, Jensen CC. Patient satisfaction and quality of life with enhanced recovery protocols. Clin Colon Rectal Surg. 2019;32(02):138–44. 30833864 10.1055/s-0038-1676480 PMC6395092

[8] Kim JW, Kim WS, Cheong J-H, Hyung WJ, Choi S-H, Noh SH. Safety and efficacy of fast-track surgery in laparoscopic distal gastrectomy for gastric cancer: a randomized clinical trial. World J Surg. 2012;36:2879–87. 22941233 10.1007/s00268-012-1741-7

[9] Wang D, Kong Y, Zhong B, Zhou X, Zhou Y. Fast-track surgery improves postoperative recovery in patients with gastric cancer: a randomized comparison with conventional postoperative care. J Gastrointest Surg. 2010;14:620–7. 20108171 10.1007/s11605-009-1139-5

[10] El-Kefraoui C, Do U, Miller A, Kouyoumdjian A, Cui D, Khorasani E, et al. Impact of enhanced recovery pathways on patient-reported outcomes after abdominal surgery: a systematic review. Surg Endosc. 2023;37(10):8043–56. 37474828 10.1007/s00464-023-10289-2

[11] Haynes AB, Haukoos JS, Dimick JB. TREND reporting guidelines for nonrandomized/quasi-experimental study designs. JAMA Surg. 2021;156(9):879–80. 33825826 10.1001/jamasurg.2021.0552

[12] Aaronson NK, Ahmedzai S, Bergman B, Bullinger M, Cull A, Duez NJ, et al. The European Organization for Research and Treatment of Cancer QLQ-C30: a quality-of-life instrument for use in international clinical trials in oncology. J Natl Cancer Inst. 1993;85(5):365–76. 8433390 10.1093/jnci/85.5.365

[13] Fitzsimmons D, Johnson C, Sandberg AA, Beger H, Birk D, Büchler M. Development of a disease specific quality of life (QoL) questionnaire module to supplement the EORTC core cancer QoL questionnaire, the QLQ-C30 in patients with pancreatic cancer. Eur J Cancer. 1999;35(6):939–41. 10533475 10.1016/s0959-8049(99)00047-7

[14] Group TE. EuroQol-a new facility for the measurement of health-related quality of life. Health Policy. 1990;16(3):199–208. 10109801 10.1016/0168-8510(90)90421-9

[15] Dolan P. Modeling valuations for EuroQol health states. Med Care. 1997;35:1095–108. 9366889 10.1097/00005650-199711000-00002

[16] Burström K, Sun S, Gerdtham U-G, Henriksson M, Johannesson M, Levin L-Å, et al. Swedish experience-based value sets for EQ-5D health states. Qual Life Res. 2014;23:431–42. 23975375 10.1007/s11136-013-0496-4 PMC3967073

[17] Daabiss M. American society of anaesthesiologists physical status classification. Indian J Anaesth. 2011;55(2):111–5. 21712864 10.4103/0019-5049.79879 PMC3106380

[18] West HJ, Jin JO. Performance status in patients with cancer. JAMA Oncol. 2015;1(7):998-. 26335750 10.1001/jamaoncol.2015.3113

[19] Dindo D, Demartines N, Clavien P-A. Classification of surgical complications: a new proposal with evaluation in a cohort of 6336 patients and results of a survey. Ann Surg. 2004;240(2):205–13. 15273542 10.1097/01.sla.0000133083.54934.ae PMC1360123

[20] Slankamenac K, Graf R, Barkun J, Puhan MA, Clavien P-A. The comprehensive complication index: a novel continuous scale to measure surgical morbidity. Ann Surg. 2013;258(1):1–7. 23728278 10.1097/SLA.0b013e318296c732

[21] Cohen J. Statistical power analysis for the behavioral sciences: Academic press; 2013. New York

[22] Andersson TK, Engström M, Bjerså K. Perceptions of experiences of recovery after pancreaticoduodenectomy—a phenomenographic interview study. Cancer Nurs. 2022;45(3):172–80. 35067575 10.1097/NCC.0000000000001021

[23] Wang D, Hu Y, Liu K, Liu Z, Chen X, Cao L, et al. Issues in patients’ experiences of enhanced recovery after surgery (ERAS): a systematic review of qualitative evidence. BMJ Open. 2023;13(2):e068910. 36810180 10.1136/bmjopen-2022-068910 PMC9945048

[24] James NE, Chidambaram S, Gall TM, Sodergren MH. Quality of life after pancreatic surgery–A systematic review. HPB. 2022;24(8):1223–37. 35304039 10.1016/j.hpb.2022.02.013

[25] Xourafas D, Pawlik TM, Cloyd JM. Independent predictors of increased operative time and hospital length of stay are consistent across different surgical approaches to pancreatoduodenectomy. J Gastrointest Surg. 2018;22:1911–9. 29943136 10.1007/s11605-018-3834-6

[26] Cai Z, Yang Y, Han Y, Fu X, Mao L, Qiu Y. Clinical validation of the comprehensive complication index in a Pancreaticoduodenectomy cohort. Eur Surg Res. 2023;64(3):334–41. 37068477 10.1159/000530634

[27] Kneuertz PJ, Pitt HA, Bilimoria KY, Smiley JP, Cohen ME, Ko CY, et al. Risk of morbidity and mortality following hepato-pancreato-biliary surgery. J Gastrointest Surg. 2012;16:1727–35. 22760965 10.1007/s11605-012-1938-y

[28] Mayhew D, Mendonca V, Murthy B. A review of ASA physical status–historical perspectives and modern developments. Anaesthesia. 2019;74(3):373–9. 30648259 10.1111/anae.14569

[29] Visser A, Geboers B, Gouma DJ, Goslings JC, Ubbink DT. Predictors of surgical complications: a systematic review. Surgery. 2015;158(1):58–65. 25731783 10.1016/j.surg.2015.01.012

[30] RCi Samverkan. National registry or tumors in the pancreatic and periampullary region. Annual report 2022 (in Swedish).

[31] Roldán J, Harrison JM, Qadan M, Bolm L, Baba T, Brugge WR, et al. Evolving trends in pancreatic cystic tumors: a 3-decade single-center experience with 1290 resections. Ann Surg. 2023;277(3):491–7. 34353996 10.1097/SLA.0000000000005142

[32] Vanier A, Oort FJ, McClimans L, Ow N, Gulek BG, Böhnke JR, et al. Response shift in patient-reported outcomes: definition, theory, and a revised model. Qual Life Res. 2021;30(12):3309–22. 33909187 10.1007/s11136-021-02846-w PMC8602159

