# Supplementary material for: Influence of an enhanced recovery programme on clinical outcomes and health-related quality of life after pancreaticoduodenectomy ad modum Whipple – an explorative and comparative single-centre study
Source: BMC Surg. 2024 Dec 21;24:407. doi: 10.1186/s12893-024-02667-x (PMC11662827; doi:10.1186/s12893-024-02667-x)
Supplement: Supplementary file 1 — Supplementary Material 1. [file 12893_2024_2667_MOESM1_ESM.docx]

Supplementary 1. Description of care content

| **Pre-ERP** | **ERP** |
| --- | --- |
| Pre-admission phase | Pre-admission phase |
| -General preoperative education/information from nurse, surgeon, and physiotherapist  -Referral to dietitian when indicated | -Individualised preoperative education/information from nurse, surgeon, and physiotherapist  -Information about smoking and alcohol cessation  -Assessment of nutritional status (modified SGA)  -Referral to dietitian when indicated  -Optimisation of pre-existing medical conditions |
| Preoperative phase | Preoperative phase |
| - Preoperative general information  - Long-term acting anxiolytics  - ≥6h fasting before surgery  - Preoperative antibiotic- and thromboprophylaxis | -Extended and ERP-related preoperative information  -No or short acting anxiolytics  -Preoperative antibiotic- and thromboprophylaxis  -Shortened fasting before surgery. No food or fluid containing fat or solid pieces after midnight the day before surgery. Preoperative carbohydrate treatment |
| Intraoperative phase | Intraoperative phase |
| -Longer acting anaesthetics and analgesics  -Liberal crystalloid fluid regimen  -Bleeding compensated 1:1 with colloids and blood products  -Non-activated TEDA  -Use of nasogastric tubing  -Occasional prevention of PONV  -Surgical site drains remaining for minimum 3 days | -Short acting anaesthetics and analgesics  -Goal directed crystalloid fluid regimen; Cardiac index 3-5 l/min/m^2^  -Bleeding compensated 1:1 with colloids and blood products  -Activated TEDA during surgery  -Use of nasogastric tubing  -Prevention of PONV  -Surgical site drains up to maximum 3 days if no leakage  -Norepinephrine in central line for mean arterial pressure above 70 mm Hg  -Cardiac output monitoring (PICCO) during surgery |
| Postoperative phase | Postoperative phase |
| -Liberal fluid regimen to achieve adequate urinary output of 2000 ml /day  - TEDA activated, and removed when tolerating oral pain medication in consultation with the patient.  -Liberal use of oral opioids for at least 3 weeks  -Use of nasogastric tube for 7 days with suction,  - Oral intake after removal of nasogastric tube. Food level incl. nutritional supplements according to the physician’s prescription.  -Extended fasting, at least during the first 5 days  -Body weight on demand when prescribed by the physician.  -P-glucose control 4-6 times/24 h  -Measurement of drain amylase days 1,3 and 5  -Observation of vital signs once every shift or as needed (after EDA removal).  -Early mobilisation (morning of the day after surgery). No specific mobilisation regimen  -Physiotherapist-prescribed and daily supervised breathing exercises with PEP. Training >8 times/day 3 sessions of 30 breaths  - Early postoperative nutritional assessment by dietician, individualised nutritional therapy during the stay; Parenteral nutrition if needed. Individualised nutritional advice before discharge and prescription of Oral Nutritional Supplement. | -Goal directed fluid regimen; Cardiac index 3-5 l/min/m2 (during stay in ICU)  -Crystalloids 1-1.5 ml/kg/h (at surgical ward)  -Early intake of fluids (POD1), solids POD5.  -Patient-controlled TEDA <POD5, thereafter opioid analgesic  -Removal of nasogastric tub (POD3 if <400ml)  - Oral intake after removal of nasogastric tube.  - Food introduction according to ERAS^®^.  -Chewing gum ≥POD1 and laxatives ≥POD2  -Body weight daily (until POD5 or as needed)  -B-glucose 4-6 times/24h  -Measurement of drain amylase POD 1, 3 and 5  -Removal of surgical site drain (POD 3 if drain amylase <35µkat/ml)  -Observation of vital signs based on NEWS (after removal of EDA)  - Tailored progressed mobilisation status but at least one-hour POD1, 2 hrs. POD2, 3 hrs. POD3 and 4 hrs. POD4.  -Physical activity by mini cycle once POD3 and ≥twice daily thereafter  -Physiotherapist prescribed and daily supervised breathing exercises with PEP. Training >8 times/day 3 sessions of 30 breaths  - Early postoperative nutritional assessment by dietician, individualised nutritional therapy during the stay; Parenteral nutrition if needed.  -Individualised nutritional advice before discharge and prescription of Oral Nutritional Supplement.  -Early preparation for discharge.  -Discharge according to specific criteria |

(Thoracal) Epidural Analgesia-(T)EDA, postoperative nausea, and vomiting -PONV, positive expiratory pressure- PEP, Subjective Global Assessment- SGA, Postoperative Day (POD)

Footnote: To measure cardiac index, Pulse Contour Cardiac Output monitoring (Pulsion) was used
